# Development of microtiter plate scale CRISPR/Cas9 transformation method for *Aspergillus niger* based on in vitro assembled ribonucleoprotein complexes

**DOI:** 10.1186/s40694-019-0066-9

**Published:** 2019-03-15

**Authors:** Joosu Kuivanen, Veera Korja, Sami Holmström, Peter Richard

**Affiliations:** 1 VTT Technical Research Centre of Finland Ltd, P.O. Box 1000, 02044 Espoo, Finland; 2 Present Address: Tampere University, Tampere, Finland; 3 Present Address: Solar Foods Ltd, Espoo, Finland

**Keywords:** *Aspergillus niger*, CRISPR, Genome editing, High-throughput, Automation, Galactarate, Mucic acid, Pectin, Galacturonate, Metabolic engineering

## Abstract

**Background:**

The CRISPR/Cas9 is currently the predominant technology to enhance the genome editing efficiency in eukaryotes. Established tools for many fungal species exist while most of them are based on in vivo expressed Cas9 and guide RNA (gRNA). Alternatively, in vitro assembled Cas9 and gRNA ribonucleoprotein complexes can be used in genome editing, however, only a few examples have been reported in fungi. In general, high-throughput compatible transformation workflows for filamentous fungi are immature.

**Results:**

In this study, a CRISPR/Cas9 facilitated transformation and genome editing method based on in vitro assembled ribonucleoprotein complexes was developed for the filamentous fungus *Aspergillus niger*. The method was downscaled to be compatible with 96-well microtiter plates. The optimized method resulted in 100% targeting efficiency for a single genomic target. After the optimization, the method was demonstrated to be suitable for multiplexed genome editing with two or three genomic targets in a metabolic engineering application. As a result, an *A. niger* strain with improved capacity to produce galactarate, a potential chemical building block, was generated.

**Conclusions:**

The developed microtiter plate compatible CRISPR/Cas9 method provides a basis for high-throughput genome editing workflows in *A. niger* and other related species. In addition, it improves the cost-effectiveness of CRISPR/Cas9 genome editing methods in fungi based on in vitro assembled ribonucleoproteins. The demonstrated metabolic engineering example with multiplexed genome editing highlights the applicability of the method.

**Electronic supplementary material:**

The online version of this article (10.1186/s40694-019-0066-9) contains supplementary material, which is available to authorized users.

## Background

Efficient and reliable genome editing is one of the cornerstones in biotechnology. Just recently, the use of programmable nucleases, especially the CRISPR/Cas9 technology [1, 2] derived from a prokaryotic immune system, has greatly improved the efficiency of targeted genome editing in eukaryotes [3, 4]. An intentionally generated double-strand break (DSB) at the chromosomal locus of interest enables high efficiency in targeted genome editing by stimulating the major DSB repair mechanisms – the homology-directed repair (HDR) and non-homologous end joining (NHEJ) pathway. In genome editing, these repair pathways mediate the insertion of exogenous DNA or generate mutations due to error-prone DSB repair into the genomic target.

The CRISPR/Cas9 is currently the predominant programmable nuclease system to generate intentional DSBs in genome editing [3, 4]. The system consists of Cas9 nuclease, typically derived from *Streptococcus pyogenes*, and the guide RNA (gRNA) components CRISPR-RNA (crRNA) and trans-activating crRNA (tracrRNA) [1, 2]. For in vivo expression, the gRNA components are often merged and called a chimeric single guide RNA (sgRNA) [2]. A 20-base protospacer sequence in the crRNA and the protospacer adjacent motif (PAM) sequence (NGG in *S. pyogenes* Cas9) at the genomic target recognized by Cas9 defines the target site guiding the binding of Cas9 to protospacer complementary sequence in the genome. The DNA endonuclease activity of Cas9 generates a DSB about 3–4 nucleotides upstream of the PAM sequence [1, 2].

In order to maintain genome integrity, the cell must repair generated DSBs. The classical NHEJ (C-NHEJ) pathway is an error-prone DSB repair mechanism requiring no or minor complementary base pairing to join the DNA-ends using blunt ligation facilitated by several accessory proteins such as the Ku70/80 complex, nucleases, polymerases and DNA ligase IV [5–7]. Due to the DNA-end processing, small genomic insertions or deletions (indels) are often generated at the breakpoint junction. Alternatively, DNA-ends at the DSB are resected generating single-stranded DNA (ssDNA) tails [5]. The Rad51 recombinase dependent HDR is a high-fidelity and error-free mechanism to recombine the resected ssDNA-ends [5]. The recombination is directed through an intact homogeneous repair DNA sequence, such as the sister chromatid but in genome editing, an exogenous repair DNA (donor DNA) flanked by homologous sequences to both sides of the DSB is typically used. In contrast, the alternative homology-based pathways to repair resected ssDNA-ends at the DSB – the single-strand annealing (SSA) and alternative end joining (alt-EJ, sometimes referred as alternative NHEJ, A-NHEJ) – are error-prone generating indels or larger chromosomal rearrangements [5–7]. For example, a recently discovered microhomology-mediated end joining (MMEJ) pathway, classified as an alt-EJ, relies on rejection and microhomology (~ 2 to 20 bases) guided complementary base paring between ssDNA-ends and a repair DNA but results in mutations at the breakpoint junction [6, 7]. In contrast to Rad51 dependent HDR, the molecular machinery in MMEJ is different by being independent of Rad51 recombinase but also of Ku70/80 complex that facilitates the C-NHEJ pathway [5–7].

In CRISPR/Cas9 genome editing, an exogenous donor DNA can be integrated into the genomic junction through different repair mechanisms. Typically HDR results in precisely joined junctions between the DSB-ends and the donor DNA while MMEJ and NHEJ pathways generate indels or substitutions at the junctions [8, 9]. In addition, different repair mechanisms may be used at different junctions of a single repairing donor DNA, such as a precise HDR mediated junction at the 5′ end and a NHEJ or MMEJ mediated junction with mutations at the 3′ end [10, 11]. However, although increasingly studied in eukaryotic cells, the knowledge on DSB repair mechanisms in the context of CRISPR/Cas9 generated DSBs is still in its early state.

Similar to other eukaryotes, CRISPR/Cas9 is becoming a standard methodology for improving the genome editing efficiency in fungi. Typically, implementation of CRISPR/Cas9 systems in fungal organisms is based on in vivo expression of Cas9 and a sgRNA molecule [12, 13]. In the fungal species with a wide selection of established tools for gene expression, such as *Saccharomyces cerevisiae*, the in vivo expression approach is the most practical choice. However, in many fungal organisms, such as filamentous fungi, tools for gene expression are poorly developed. In addition, construction and cloning of DNA vectors used in the in vivo CRISPR/Cas9 approach can be time consuming and labour intensive. Thus, implementing Cas9 and the gRNA components as an in vitro assembled ribonucleoprotein (RNP) complex in transformation may be a viable alternative, especially for high-throughput (HTP) approaches.

The filamentous fungus *Aspergillus niger* is one of the most widely used production platforms for enzymes and chemicals in industrial biotechnology [14]. A few approaches of CRISPR/Cas9 facilitated genome editing in *A. niger* have been reported. In the pioneering work in filamentous fungi gene deletions were carried out using in vivo expression of Cas9 and sgRNA from a plasmid and C-NHEJ-pathway mediated error-prone repair of the generated DSB [15]. Subsequently, approaches based on HDR mechanism using the integrating donor DNA and plasmid based Cas9 expression together with co-transformed in vitro synthesized sgRNA have been reported [16, 17]. In recent studies, short 40-bp flanking sequences as part of donor DNA [18, 19] or oligo nucleotides [20] were used as donor DNA together with in vivo expressed Cas9 and sgRNAs, resulting in efficient genome editing in *A. niger*. For in vivo sgRNA expression in *A. niger*, RNA polymerase II promoter together with self-processing ribozyme sequences was first reported [15]. After that, a RNA polymerase III promoters U3 [20], U6 [18] and S5 [19] have been used for sgRNA expression in *A. niger*. However, no reports on genome editing in *A. niger* based on in vitro assembled RNP complexes exist to date.

In few other fungi, the use of in vitro assembled RNP complexes in CRISPR/Cas9 have been reported. The filamentous fungi *Penicillium chrysogenum* [21], *Aspergillus fumigatus* [22] and *Mucor circinelloides* [23], and the yeast species belonging to *Candida* [24] were engineered using in vitro assembled RNP complexes in CRISPR/Cas9 system. However, transformation methods reported for filamentous fungi in general require relatively large reaction volumes, which is challenging for HTP workflows.

In the present study, we developed a CRISPR/Cas9 facilitated transformation and genome editing method for *A. niger*, which is based on in vitro assembled RNP complexes and downscaled transformation reaction volumes. The smaller reaction volumes enable the use of 96-well microtiter plates (MTPs) for HTP workflows. In addition, the use of top-agar is not required in the plating after the transformation facilitating the workflow with multiple reactions. The method was optimized using the NHEJ proficient background *∆pyrG A. niger* strain, where targeted genome editing has required extensive genotype screening. In addition, the use of a liquid handling robot was demonstrated for the workflow. In the end, the developed method was used for multiplexed genome editing in a metabolic engineering application. As a result, an *A. niger* strain was generated for the improved production of galactarate, a potential platform chemical. The strain was engineered by simultaneously deleting three competing pathway genes and one regulatory gene by replacing them with expression cassettes for a heterologous gene.

## Results

### Down-scaled transformation reaction for *A. niger*

We aimed to develop a fast and robust CRISPR/Cas9 genome editing method for *A. niger* which is based on in vitro assembled RNP complexes. Another requirement was to downscale the reaction volume in the transformation reaction to be compatible with MTPs. Typically, transformation reactions in filamentous fungi using protoplasts are carried out in relatively large volumes (> 1 ml) using e.g. 15-ml tubes. Downscaling the transformation volume below 200 µl would enable the use of regular MTPs, which are compatible with multichannel pipettes and liquid handling robots. In addition, the downscaled reaction volume would require less protoplasts, RNP complex and donor DNA to be used in the transformation which will decrease the cost per transformation in our case by approximately 90%.

**Fig. 1:**
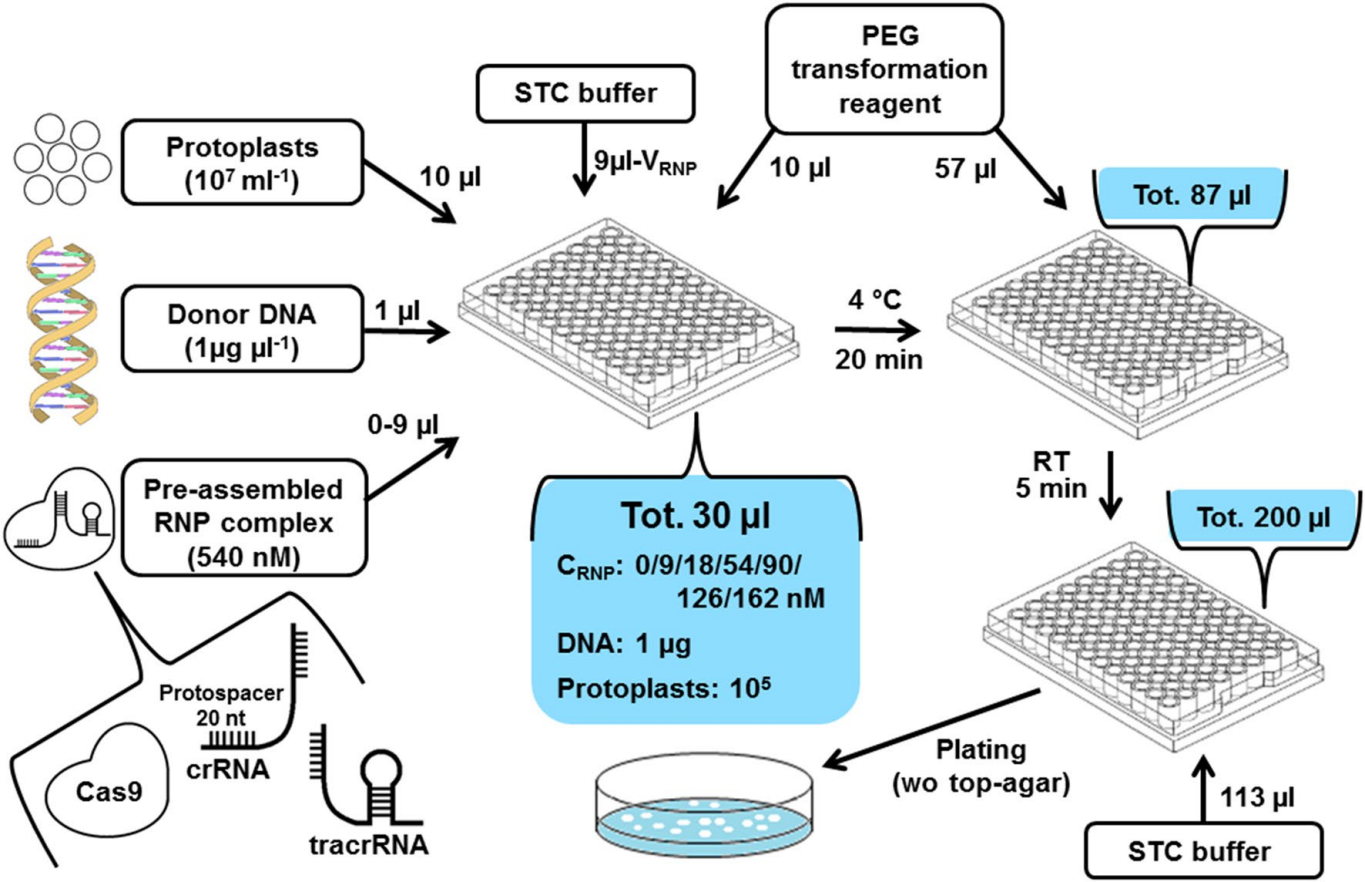
Schematic presentation of the developed microtiter plate scale CRISPR/Cas9 transformation method for *A. niger* which is based on the transformation of in vitro assembled RNP complex

Protoplasts were generated from a 125-ml overnight shake flask pre-cultivation using the pyrimidine-deficient *A. niger ∆pyrG* strain derived from ATCC1015 wild type strain. We standardized the concentration of the protoplast working solution to 10^7^ protoplasts ml^−1^ and 10 µl of this working solution was used in a single transformation resulting in 10^5^ protoplasts per 30-µl transformation reaction (Fig. 1). The concentration was based on the comparison of colony-forming units (CFUs) between the transformation reactions carried out using 10^5^ or 10^6^ protoplast together with 1 µg of donor DNA and varying concentrations of RNP complexes for a gene deletion (*gaaX*, details in the next paragraphs) (Additional file 1: Fig. S1). The higher protoplast concentration resulted in too many colonies for single colony isolation. In addition, the use of lower protoplast concentration enabled generation of several hundred of transformation reactions from a single 125-ml pre-cultivation.

Due to the use of in vitro assembled RNPs without direct selective pressure towards its uptake in the cell, a selection marker had to be used in the donor DNA. In order to establish a standardized and modular workflow for the donor DNA construction, we applied the modular cloning (MoClo) tool kit developed for yeast [25] that is based on the Golden Gate cloning method and standardized building blocks with specific sticky ends for each of the building block types. Flanking sequences for *A. niger* genomic targets and the *pyrG* gene from *Aspergillus oryzae*, encoding a orotidine-5-phosphate decarboxylase to be used as a selection marker, were generated and used in the MoClo system. We standardized the concentration of donor DNA stock solution for the *A. niger* transformations to 1 µg µl^−1^ and 1 µg of the donor DNA was used in a single transformation reaction (Fig. 1). In addition, to increase the fluency of the workflow protoplasts were plated onto selective agar plates without addition of typically used liquid top-agar. Details of the developed transformation method and workflow are schematically described in Fig. 1.

### Optimizing the Cas9-crRNA-tracrRNA RNP concentration in genome editing

In order to develop a high-fidelity transformation method but also to increase the cost-efficiency, we started with the optimization of the RNP concentration used in the protocol. We hypothesized that the RNP concentration is the limiting factor in targeting efficiency in the presence of the standardized protoplast (10^5^ in 30 µl) and donor DNA (1 µg in 30 µl) concentrations. For the optimization, *A. niger gaaX* was selected as a target gene for deletion using a donor DNA containing the 1500-bp flanking sequences and *pyrG* selection marker to replace the whole *gaaX* open reading frame (ORF) via HDR. The target gene *gaaX* is a recently discovered repressor protein regulating pectin and d-galacturonate metabolism in *A. niger* [26]. Deletion of *gaaX* has shown to relieve the repression of pectin and d-galacturonate associated genes in the presence of more preferred carbon sources in *A. niger*. Pectin consists mainly of d-galacturonate but also contains other monomers that are more preferred carbon sources for *A. niger*. Thus, the deletion could be beneficial in *A. niger* strains engineered for the production of chemicals from d-galacturonate in a consolidated bioprocess using pectin rich biomass as raw material.

**Fig. 2:**
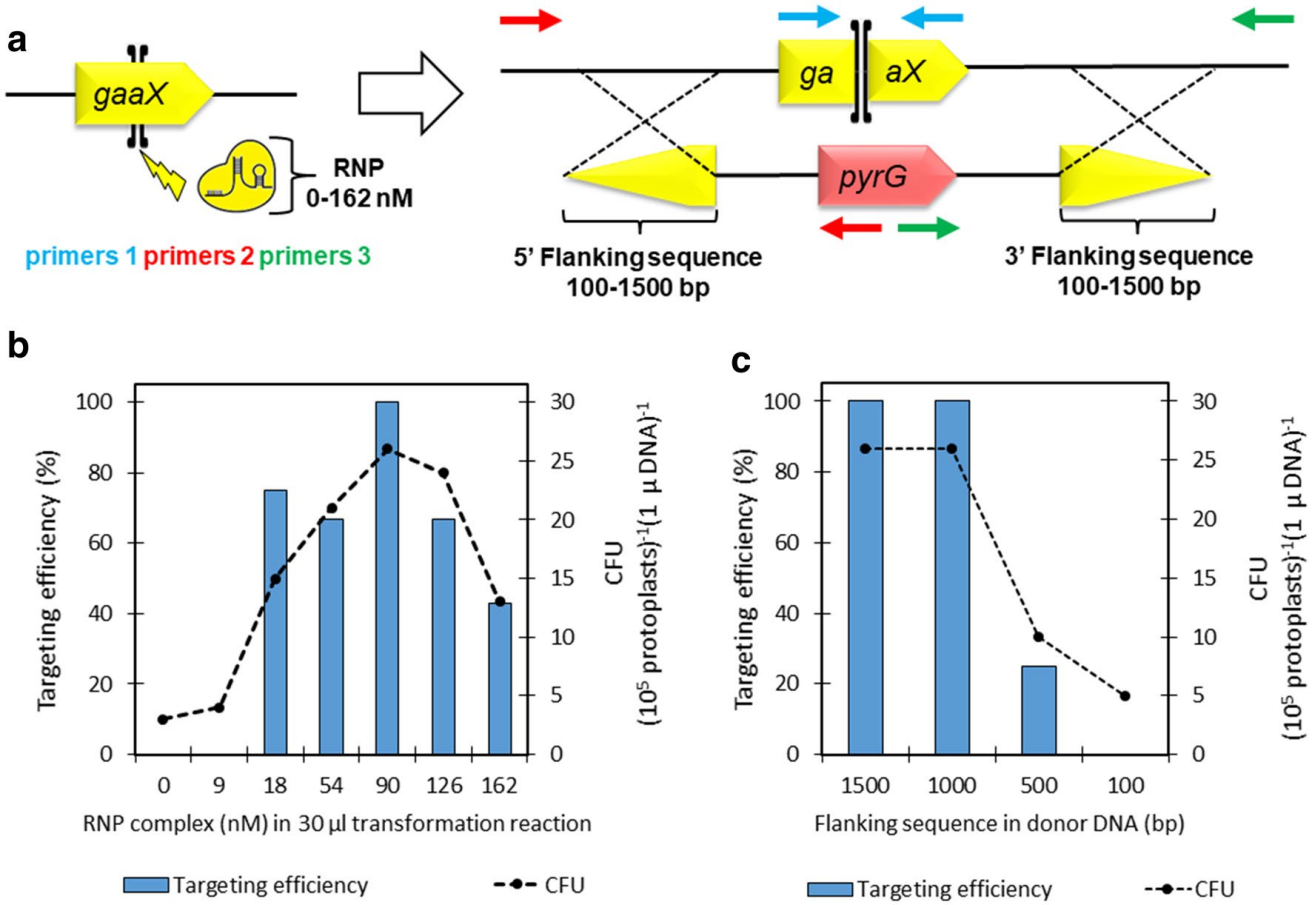
**a** CRISPR/Cas9 facilitated deletion of *A. niger gaaX* gene based on in vitro assembled RNP complex and donor DNA containing *pyrG* selection marker. Targeting efficiency was analysed by using colony PCR ensuring the absence of *gaaX* ORF (primers 1, oPEEL-294/295) and the presence of donor DNA cassette at the correct genomic locus (primers 2, oPEEL-279/292 and 3, oPEEL-293/334). Targeting efficiency and the colony forming units (CFU) per 10^5^ protoplasts and 1 µg donor DNA **b** with 1500 bp flanking sequence in the donor DNA and different concentrations of RNP complex and **c** with 90 nm RNP complex and varying lengths of flanking sequence in the donor DNA. 3–12 colonies from each transformation depending on colony number on plates were analyzed

In the RNP complex assembly, a chemically synthesized crRNA containing a 20-bp protospacer sequence for *gaaX* (gERA-001, Additional file 1: Table S1) was first hybridized with tracrRNA and then assembled with Cas9 protein in vitro (Fig. 1 and Additional file 1: Fig. S2). The resulting RNP complex should generate a single DSB at *gaaX* ORF, which is subsequently repaired by the HDR mechanism using the donor DNA (Fig. 2a). Several RNP concentrations were tested in the transformation reactions carried out in MTPs together with the standardized protoplast and donor DNA concentrations. The resulting CFUs were calculated and the targeting efficiency was analyzed using colony PCR (Fig. 2a, b and Additional file 1: Figs. S1 and S3). The criteria for the correct insertion of donor DNA were correctly sized amplification products from the 5′ and 3′ breakpoint junctions and the absence of the *gaaX* ORF (Fig. 2a). Transformations with donor DNA but without or with 9 nM RNP resulted only in a few colonies, all without correct donor DNA insertion at the genomic target (Fig. 2b and Additional file 1: Figs. S1 and S3). The observed low CFUs without RNP indicate that the background is most likely resulting from random ectopic genomic integrations of donor DNA. Between the RNP concentrations 18 and 90 nM, CFUs gradually increased and the majority of the colonies had the correctly inserted donor DNA. Notably, all of the colonies generated with 90 nM RNP had the correct donor DNA insertion. RNP concentrations 126 and 162 nM resulted in a decline in CFU but also in the targeting efficiency. Thus, we decided to use a concentration of 90 nM RNP in the following experiments.

Next, we investigated whether the shorter 5′ and 3′ flanking sequences are functional for the *gaaX* gene deletion using the developed transformation method. Four different flanking sequence lengths, 1500, 1000, 500 and 100 bp, were tested together with 90 nM RNP (Fig. 2c, Additional file 1: Fig. S3). As a result, we observed that 1500- and 1000-bp flanking sequences both resulted in similar CFUs as well as 100% targeting efficiency (all tested colonies with correct donor DNA insertion). Both, the CFU and targeting efficiency decreased when 500-bp flanking sequences were used and no correct colonies were observed with the donor DNA containing 100-bp flanking sequences. Due to decreased CFUs with the decreasing flanking sequence lengths, we conclude that most likely the predominant DSB repair mechanism in our transformations was Rad51 dependent high-fidelity HDR, requiring relatively long homology sequences. In order to confirm the functionality of the transformation method and workflow, deletion of another target locus from the NHEJ proficient *A. niger ∆pyrG* strain was tested. We selected the polyketide synthase gene *albA* which upon deletion results in a phenotype of disrupted conidia pigment formation [15]. The RNP concentrations 0, 90 and 162 nM were tested together with donor DNA containing 1000-bp flanking sequences and the *pyrG* marker. Although much lower CFU values were obtained, the phenotype of the resulting colonies was 100% correct (Additional file 1: Fig. S4).

### RNP CRISPR/Cas9 facilitated gene deletion using liquid handling robot

As a proof-of-concept, the developed transformation method was transferred to Beckman Coulter Biomek NX liquid handling robot. Deletion of *A. niger gaaX* was demonstrated using the same protocol (Fig. 1) with 1500-bp flanking sequences in the donor DNA and using the RNP concentrations 162, 90, 54, 18, 9 and 0 nM. The transformation reactions were carried out in 96-well MTP containing reservoir wells for protoplasts, donor DNA and RNP, and with separate reservoir plates for PEG transformation solution and STC buffer (Additional file 1: Fig. S5). All the components for each transformation reaction were combined to single wells followed by shaking for 10 s. The incubation steps at + 4 °C and RT were also carried out on the robot deck while the plating was implemented as a manual step. A similar level of CFU values (Table 1; Additional file 1: Fig. S6) as with the manual transformation execution (Fig. 2b) was obtained. A few colonies from each transformation reaction were randomly picked and tested for the correct donor DNA insertion using colony PCR. The analysis revealed the correct donor DNA insertion for most of the tested colonies when the RNP concentration 162–54 nM was used, although 100% efficiency was not achieved (Table 1; Additional file 1: Fig. S3). The proportion of the negative colonies increased with the lowering RNP concentration. Nevertheless, we conclude that the developed transformation method is suitable for the implementation using liquid handling robots.

**Table 1:** Colony forming units (CFU) and deletion efficiencies of the *gaaX* gene using a liquid handling robot to carry out the transformation

RNP (nM)	CFU	Colonies screened	Correct *ΔgaaX*
162	25	5	4
90	28	3	2
54	15	2	1
18	10	4	2
9	13	6	2
0	6	5	1

### Multiplexed RNP CRISPR/Cas9 for rapid strain engineering in *Aspergillus niger*

**Fig. 3:**
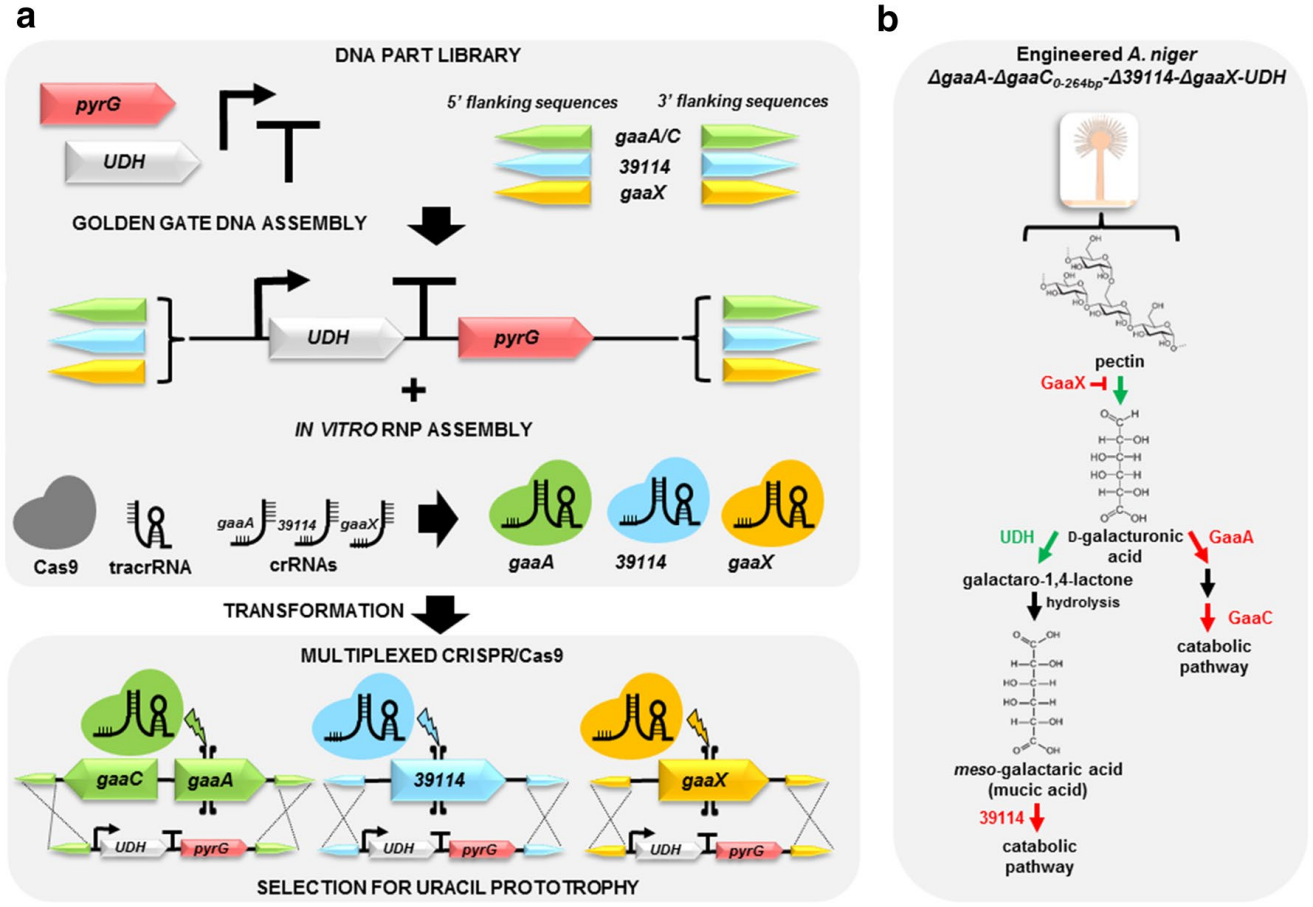
Schematic presentation of the **a** workflow and **b** target genes for multiplexed *A. niger* genome editing using the microtiter plate scale CRISPR/Cas9 method. The workflow was demonstrated by engineering the fungal d-galacturonate pathway for galactarate (mucic acid) production. The competing pathway genes *gaaA*, encoding a d-galacturonate reductase, *gaaC*, encoding an l-galactonate dehydratase, and *39114*, encoding an enzyme involved in galactarate catabolism, and *gaaX*, encoding a repressor protein of pectin catabolic pathways, were deleted by replacing the genes with DNA cassettes expressing the bacterial uronate dehydrogenase (UDH)

As a metabolic engineering application and in order to test multiplexed genome editing, we decided to use the method for engineering *A. niger* strains for galactarate production (Fig. 3). Galactarate, also known as mucic acid, is a dicarboxylic acid that can be converted further to adipic acid or furandicarboxylic acid (FDCA) [27]. The production using d-galacturonate or its polymer pectin as substrate has been demonstrated in the engineered *A. niger* strain in which the first d-galacturonate pathway gene, *gaaA*, was deleted and a bacterial uronate dehydrogenase (UDH) expressed [28]. Recently, the product titer and yield was improved by deleting a competing pathway, the gene *39114* (protein ID 39114, JGI MycoCosm, A. niger ATCC 1015 v.4.0 database) involved in the catabolism of galactarate [16]. In the genome of *A. niger*, *gaaA* is clustered with the third gene in the d-galacturonate pathway, *gaaC* encoding the 2-keto-3-deoxy-l-galactonate aldolase [29]. Although deletion of *gaaA* should block most of the carbon flux toward the pathway, *ΔgaaA* strain can still slowly catabolize d-galacturonate [30]. Thus, simultaneous deletion of another gene form of the d-galacturonate pathway, such as *gaaC,* catalyzing the third reaction could be beneficial.

We decided to generate three *A. niger* strains *ΔgaaA*-*ΔgaaC*_*0*–*264bp*_-*UDH*, *ΔgaaA*-*ΔgaaC*_*0*–*264bp*_-*Δ39114*-*UDH* and *ΔgaaA*-*ΔgaaC*_*0*–*264bp*_-*Δ39114*-*ΔgaaX*-*UDH* using the transformation method by manual pipetting (Fig. 3a, b). In addition to the *gaaX* crRNA (gERA-001) that was used in the method optimization, two crRNAs (Additional file 1: Table S1) for each of the target genes were designed and RNPs assembled in vitro. All the donor DNA cassettes for the target genes contained 1500-bp flanking sequences, *pyrG* selection marker and the *UDH* gene under the control of d-galacturonate inducible *gaaB* promoter (*P*_*gaaB*_) from *A. niger* and were constructed following the standardized MoClo protocol (Fig. 3a). In all of the strains, the *gaaA*-*gaaC* gene cluster was replaced with the UDH expression cassette deleting the *gaaA* ORF, 264 bp of the *gaaC* ORF starting from the start codon and the bidirectional promoter region of *gaaA* and *gaaC*. For the strains *ΔgaaA*-*ΔgaaC*_*0*–*264bp*_-*Δ39114*-*UDH* and *ΔgaaA*- *ΔgaaC*_*0*–*264bp*_-*Δ39114*-*ΔgaaX*-*UDH*, multiplexed genome editing using the method was tested with two or three genomic targets, respectively. Simultaneous deletion of the *A. niger* genes *gaaA*-*gaaC* and *39114* and replacing them with UDH expression cassettes was implemented as a two-target demonstration and a similar approach for *gaaA*-*gaaC*, *39114* and *gaaX* as a three-target demonstration. Notably, the copy number of targeted UDH expression cassettes increased with the increasing target gene number in the resulting strains. Despite the multiple targets and donor DNAs, only single selection for uracil prototrophy was used.

From the transformations (Additional file 1: Fig. S7) with the single genomic target (the strain *ΔgaaA*-*ΔgaaC*_*0*–*264bp*_-*UDH*), all the screened colonies generated using a single or two different crRNAs in combination had the correct donor DNA insertion (Table 2; Additional file 1: Fig. S8). Depending on crRNA combinations, the two-target multiplexing (*ΔgaaA*-*ΔgaaC*_*0*–*264bp*_-*Δ39114*-*UDH*) resulted in targeting efficiencies of 0, 13 or 25%. In the three-target multiplexing (the strain *ΔgaaA*-*ΔgaaC*_*0*–*264bp*_-*Δ39114*-*ΔgaaX*-*UDH*), the correct donor DNA insertion for each of the genomic targets was observed in 1 out of 17 screened colonies including all three different RNP combinations (Table 2; Additional file 1: Fig. S9). We also sequenced all the breakpoint junctions from the selected *ΔgaaA*-*ΔgaaC*_*0*–*264bp*_-*Δ39114*-*UDH* and *ΔgaaA*-*ΔgaaC*_*0*–*264bp*_-*Δ39114*-*ΔgaaX*-*UDH* strains (Additional file 1: Fig. S10). As a result, no indels or mutations were observed in the junctions indicating the high fidelity HDR as the repair mechanism for the DSBs.

**Table 2:** Efficiencies of correct replacements of target genes with the *UDH* expression cassette in multiplexed CRISPR/Cas9 using RNP complexes

Target genes	gRNA	Colonies screened	Correct *ΔgaaA*	Correct *Δ39114*	Correct *ΔgaaX*	All correct	Targeting efficiency (%)
*gaaA*	gERA-009	2	2	–	–	2	100
	gERA-010	5	5	–	–	5	100
	gERA-009 + gERA-010	5	5	–	–	5	100
*gaaA* + *39114*	gERA-009 + gERA-015	4	3	1	–	1	25
	gERA-010 + gERA-016	7	1	2	–	0	0
	gERA-009 + gERA-015+	8	1	3	–	1	13
	gERA-010 + gERA-016						
*gaaA* + *39114* + *gaaX*	gERA-001 + gERA-009 + gERA-015	6	3	1	2	0	0
	gERA-002 + gERA-010 + gERA-016	6	1	1	4	1	17
	gERA-001 + gERA-009 + gERA-015 + gERA-002 + gERA-010 + gERA-016	5	2	2	2	0	0

Two different crRNAs for each of the target genes were designed and tested in different combinations

In order to investigate the copy numbers of integrated *UDH* expression cassettes, qPCR analysis of the engineered strains *ΔgaaA*-*ΔgaaC*_*0*–*264bp*_-*UDH*, *ΔgaaA*-*ΔgaaC*_*0*–*264bp*_-*Δ39114*-*UDH* and *ΔgaaA*-*ΔgaaC*_*0*–*264bp*_-*Δ39114*-*ΔgaaX*-*UDH* was carried out. Although the breakpoint junctions of the targeted *UDH* expression cassette integrations were confirmed to be HDR generated, the copy number analysis revealed several additional copies of the *UDH* cassettes being integrated into the genome in all of the analyzed strains (Additional file 1: Fig. S11).

The resulting strains *ΔgaaA*-*ΔgaaC*_*0*–*264bp*_-*UDH*, *ΔgaaA*-*ΔgaaC*_*0*–*264bp*_-*Δ39114*-*UDH* and *ΔgaaA*-*ΔgaaC*_*0*–*264bp*_-*Δ39114*-*ΔgaaX*-*UDH* were tested for galactarate production and compared to the recently developed production strain M1767. Similar to the strain generated through two-target multiplexing, M1767 has the genotype *ΔgaaA*-*Δ39114*-*UDH*, however, without the deletion of *gaaC*. In addition, UDH in M1767 was expressed from an expression cassette under the widely used *P*_*gpdA*_ instead of *P*_*gaaB*_ and with an unknown copy number of integrated cassettes in genome.

**Fig. 4:**
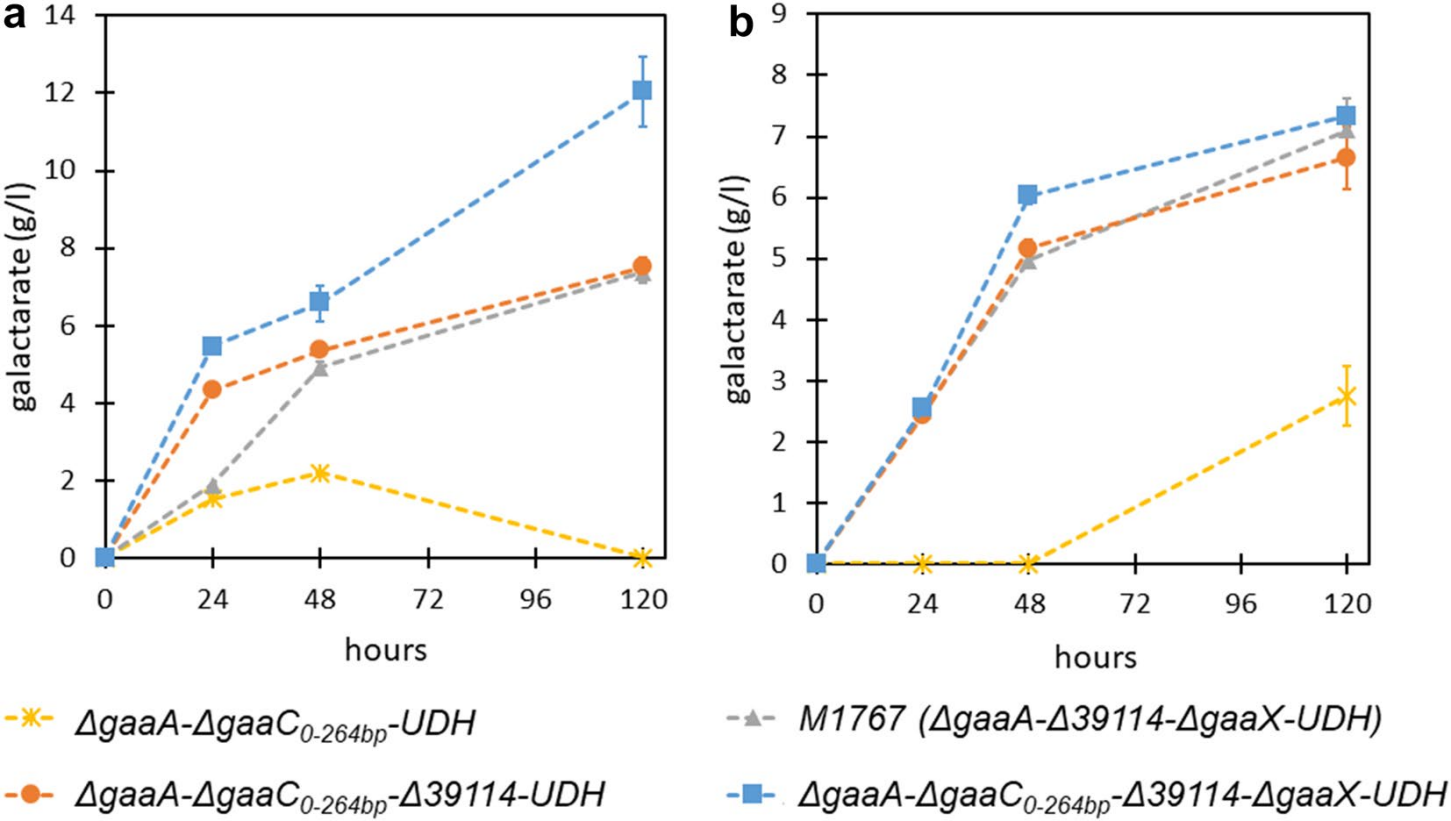
Production of galactarate from **a** 20 g l^−1^ pectin and **b** 20 g l^−1^ d-galacturonate with the engineered *A. niger* strains generated using the multiplexed microtiter plate scale CRISPR/Cas9 genome editing method. The engineered strains resulting from multiplexed (blue squares and yellow circles) and single target (red diamonds) CRISPR genome editing were cultivated on YP medium supplemented with 20 g l^−1^ pectin or d-galacturonate. The galactarate producing strain from the previous study [16] (grey triangles) was used as a control strain. Values represent the means of three biological replicates ± SD

The cultivations were carried out in 24-well plates using 4-ml cultivation volume and 20 g l^−1^ pectin (Fig. 4a) or d-galacturonate (Fig. 4b) as carbon source. As observed earlier [28], the strain *ΔgaaA*-*ΔgaaC*_*0*–*264bp*_-*UDH* generated in this study did produce galactarate, but however, also catabolized the product (Fig. 4a). Deletion of *39114* resulted in improved production performance by the strain *ΔgaaA*-*ΔgaaC*_*0*–*264bp*_-*Δ39114*-*UDH* similar as with the strain M1767 described recently [16] both on pectin and d-galacturonate. A similar final galactarate concentration was observed with the *ΔgaaA*-*ΔgaaC*_*0*–*264bp*_-*Δ39114*-*UDH* strain generated herein as with the strain M1767, however, a higher galactarate concentration was observed after 24 h on pectin cultivation while the performance of the strains was similar when d-galacturonate was used as carbon source. This may arise from the differences between the strains, such as *gaaC* deletion and *P*_*gaaB*_ controlling the *UDH* expression instead of *P*_*gpdA*_ in the *ΔgaaA*-*ΔgaaC*_*0*–*264bp*_-*Δ39114*-*UDH* or from the possible difference in copy numbers of *UDH* expression cassettes in the strains. The strain *ΔgaaA*-*ΔgaaC*_*0*–*264bp*_-*Δ39114*-*ΔgaaX*-*UDH* resulting from the three-target multiplexing had clearly improved production capacity when pectin was used as carbon source. A product titer of around 12 g l^−1^ was observed showing an improvement of about 60% in the pectin cultivations when compared to the strains *ΔgaaA*-*ΔgaaC*_*0*–*264bp*_-*Δ39114*-*UDH* and M1767 without the *gaaX* deletion.

## Discussion

After the emergence of the CRISPR/Cas9 technology, efficiency in genome editing has dramatically improved. The molecular constituents in the CRISPR/Cas9 genome editing, Cas9 and gRNA, are often implemented as in vivo expressed DNA constructs but can also be transformed as an in vitro assembled RNP complex. The latter approach is widely used in animal cells [31–34], but however, much less used for fungal organisms. The approach of using in vitro assembled RNP complexes provides a robust, cloning-free and HTP compatible way to implement CRISPR/Cas9. In addition, it eliminates the risk of unwanted genomic integrations of DNA constructs expressing Cas9 and sgRNA. In this study, we described a genome editing and transformation method for *A. niger*, which relies on in vitro assembled RNP complexes, integrating donor DNA with flanking sequences and downscaled reaction volumes suitable for MTP workflows. A targeting efficiency of 100% was observed for a single target while multiplexing with two and three targets resulted in successful transformations with lower targeting efficiencies.

In the first report of using in vitro assembled RNPs in CRISPR/Cas9 genome editing for filamentous fungi, Pohl et al. [21] deleted the *pks17* gene from a NHEJ deficient *P. chrysogenum* strain using *amdS* as a selection marker and 1000-bp flanking sequences in the donor DNA. Different RNP concentrations were tested in the transformation reaction ranging from 45 to 1520 nM. As a result, 110 nM RNP concentration provided the highest CFU while higher RNP concentrations gradually decreased the CFU. This is a similar observation as in the present study where the optimal RNP concentration was 90 nM and higher concentrations decreased the CFU. The CFU decline with the higher RNP concentrations in our study may have partially been the result of the lower concentration of osmotic buffer in the transformation reaction (less STC buffer added) compromising the protoplast viability. This could be avoided by using higher concentration of tracr/crRNA and Cas9 components in the assembly reaction or using osmotic buffer in the assembly reaction. However, we do not see the need to use higher RNP concentration than 90 nM in the method. Shortening of the flanking sequences in the donor DNA up to 60 bp resulted still in correct gene deletion in *P. chrysogenum*, however, with decreasing CFUs [21]. We also observed decreasing CFU with flanking sequences below 1000-bp in length while the donor DNA with 100-bp flanking sequences resulted in no correct colonies. In addition, the RNP CRISPR/Cas9 was multiplexed in *P. chrysogenum* for two genes using the same *amdS* selection marker in both donor DNAs [21]. This resulted in 4 out of 8 tested clones with both target genes deleted.

In *A. fumigatus*, in vitro assembled Cas9-gRNA RNPs were recently used in deleting the polyketide synthase gene *pksP* [22]. Cas9 stock solution of 1 µg µl^−1^ corresponding to 6100 nM concentration was used in the RNP assembly and a final RNP concentration of about 100 nM was used in the transformation reaction. Two different RNPs were simultaneously used cleaving the whole gene together with donor DNA containing an antibiotic marker and short 35- or 50-bp flanking sequences. As a result, 100% deletion efficiency was reported in a NHEJ deficient strain whereas efficiencies of 40% and 74% were observed with the NHEJ proficient wild type strain. Interestingly, increased amounts of repairing donor DNA decreased the targeting efficiency in wild type, most likely due to ectopic integrations through NHEJ-mediated repair. In our approach, 100% targeting efficiency was observed for a single gene in a NHEJ proficient *A. niger* strain. In contrast, we used longer flanking sequences to stimulate HDR mechanism and adjusted the reaction conditions so that only very few colonies were obtained without RNP through the NHEJ-mediated ectopic integrations. Use of long flanking sequences is a trade-off between more laborious donor DNA construction and higher frequency of correct genomic integration, however, donor DNA construction can be significantly accelerated by using modular cloning systems as we used the MoClo system [25]. However, despite the high targeting efficiency, the copy number analysis revealed ectopic integrations in the strains obtained from the RNP CRISPR transformations. Thus, we conclude that it may be a difficult task to avoid completely ectopic donor DNA integrations in a NHEJ proficient strain.

In the previous studies with *P. chrysogenum* [21] and *A. fumigatus* [22] close to similar RNP concentrations (90–100 nM) as in our study were used in the transformation reaction. In contrast, we downscaled the reaction volume to 30 µl compared to > 250 µl [21, 22] used in the initial incubation phase. This reduces the absolute amount of RNP needed per transformation almost tenfold. We have calculated that the price of RNP complex per reaction is less than 1 euro, when only a single crRNA stock is repeatedly used until completion. Obviously, if a crRNA is used only once the cost per transformation increases up to ~ 60 to 70 euro. This cost may possibly be lower when a large number of crRNA molecules are purchased from a supplier in a MTP format. Nevertheless, the use of outsourced chemically synthesized crRNA and tracrRNA molecules is justified due to savings in labor costs required for cloning or in vitro sgRNA synthesis.

The final volume after the addition of STC buffer remains below 200 µl in our method. This is crucial in HTP workflows where MTPs together with liquid handling automation is used. As we demonstrated in the study, although still needing some optimization such as choosing an optimal liquid class for the viscous PEG containing transformation reagent, our method is compatible with liquid handling robots speeding up the preparation of transformation reactions. If large numbers of crRNAs would be provided in MTP format, even the RNP assembly reactions would be possible to implement using a liquid handler. Plating onto selective agar plates is a bottleneck in our system; however, we showed that colonies can be obtained without traditionally used addition of melt top-agar. With a suitable plating device such as spiral plater in a robotic system it could be possible to automate the transformation workflow completely. Nonetheless, even without liquid handling automation, the developed MTP compatible transformation method enables the use of multi-channel pipettes and implementation of about one hundred simultaneous transformation reactions is possible according to our experience.

In the metabolic engineering application, we multiplexed our in vitro assembled RNP CRISPR/Cas9 method with two and three target genes. Notably, we used relatively long donor DNA containing selection marker and expression cassette for UDH comprising ~ 4300-bp sequence in addition to ~ 1500-bp flanking sequences generating a donor DNA cassette of ~ 7300-bp. Multiplexing and using only single selection pressure decreased the targeting efficiency, however, it was possible to obtain correct clones using relatively small screening effort. We used the method for generating engineered *A. niger* strains capable of hydrolyzing pectin to d-galacturonate and to oxidize it to galactarate. Galactarate is a bulk chemical that can be chemically converted to the plastic monomers adipic acid and FDCA [27]. For the first time, we tested the effects of deleting the pectin and d-galacturonate pathway repressor gene *gaaX* in the galactarate producing *A. niger* strain. In the strain, three genomic targets were deleted by replacing them with UDH expression cassettes in a single transformation. The resulting strain *ΔgaaA*-*ΔgaaC*_*0*–*264bp*_-*Δ39114*-*ΔgaaX*-*UDH* produced about 60% higher concentration of galactarate from pectin after 5 days than the strain *ΔgaaA*-*ΔgaaC*_*0*–*264bp*_-*Δ39114*-*UDH* without *gaaX* deletion. However, varying copy numbers of *UDH* expression cassettes between the strains may have had an effect on galactarate production. Nevertheless, this example shows how relatively complicated genome editing tasks for metabolic engineering can be rapidly carried out in *A. niger* using the method developed herein.

## Conclusions

In this study, we developed a MTP compatible CRISPR/Cas9 genome editing method for the filamentous fungus *A. niger*. The method is based on in vitro assembled RNP complexes and repairing donor DNA that contains a selection marker enabling robust and cloning-free implementation of Cas9 and gRNA. Due to downscaled volumes in transformation reactions, the cost per transformation is reduced. The method and workflow provide a basis for HTP genome editing approaches in *A. niger* and related species. Beyond that, the method may be applicable to many other filamentous fungi where protoplasts can be obtained.

## Methods

### Strains, media and culture conditions

The *Aspergillus niger* strain ATCC 1015 (D-081297, VTT Culture Collection; CBS 113.46) was used as a wild type. The pyrimidine-deficient *A. niger* strain *∆pyrG* (deleted orotidine-5′-phosphate decarboxylase) derived from ATCC 1015 [28] was used as a parental strain. The engineered galactarate producing strain M1767 *∆gaaA*-*∆39114*-*UDH* containing the *UDH* gene from *Agrobacterium tumefaciens* was described previously [16]. Engineered *A. niger* strains generated in this study are listed in Additional file 1: Table S1. All the plasmids were produced in *Escherichia coli* TOP10 cells.

Luria Broth culture medium supplemented with 100 µg ml^−1^ of ampicillin or 25 µg ml^−1^ chloramphenicol and culture conditions of 37 °C and 250 rpm were used for *E. coli* cultures. *A. niger* spores were generated on potato-dextrose plates. Spores were inoculated to 125 ml of YP medium (10 g l^−1^ yeast extract, 20 g l^−1^ peptone) containing 30 g l^−1^ gelatin for pre-cultivations. Mycelia were pre-grown in 250-ml Erlenmeyer flasks by incubating overnight at 28 °C, 200 rpm and harvested by vacuum filtration, rinsed with sterile water and weighed. In *A. niger* transformations, plates containing uracil deficient synthetic complete medium (SC-URA, 6.7 g l^−1^ yeast nitrogen base) supplemented with 20 g l^−1^ d-glucose, 1.2 M d-sorbitol and 20 g l^−1^ agar were used. In galactarate production, YP medium supplemented with 20 g l^−1^ citrus pectin (Sigma) was used and cultivations were carried out in 24-well plates in 4-ml volume at 28 °C and 800 rpm. 7.5 g l^−1^ (wet) of pre-cultivated mycelium was used for inoculation.

### DNA construction

All the DNA cassettes were constructed using the MoClo tool kit system [25] based on Golden Gate cloning. Different flanking sequences and *P*_*gaaB*_ were PCR amplified (KAPA Hifi PCR Kit, KAPA Biosystems) from *A. niger* ATCC105 genomic DNA and cloned into the entry vector pYTK001. The selection marker *pyrG* was amplified from *Aspergillus oryzae* (D-88349, VTT Culture Collection) genomic DNA and *UDH* (*Agrobacterium tumefaciens*) from previously described expression cassette [28]. All the primers are listed in Additional file 1: Table S1. For the *gaaX* and *albA* deletion cassettes without *UDH*, in addition to flanking sequences the following MoClo entry vectors were used: pYTK002, 048, 067 and 089. In the *udh* expression cassettes, in addition to flanking sequences, *A. niger P*_*gaaB*_ and *Saccharomyces cerevisiae ENO1* terminator (pYTK051) the following MoClo entry vectors were used: pYTK002, 051, 067 and 089.

### CRISPR/Cas9 and *A. niger* transformations

All the protospacer sequences (Additional file 1: Table S1) in crRNAs were designed and screened against off targets using Geneious software and confirmed with the online tool CHOPCHOP [35, 36]. Cas9, tracrRNA and crRNA were purchased (IDT) and the RNP assembly was carried out following the manufacturer’s instructions as described in the Additional file 1: Fig. S2. The downscaled *A. niger* protoplast transformation reactions were carried out in 96-well plates and the protocol is described in the Fig. 1 in detail. STC buffer contained 10 mM Tris–HCl, 1.33 M sorbitol and 50 mM CaCl_2_ (pH 8.0). The PEG solution contained 25% PEG 6000, 50 mM CaCl_2_ and 10 mM Tris–HCl (pH 7.5). Protoplasts were generated in KMC buffer containing 1 M KCl, 25 mM CaCl2, 10 mM Tris–HCl and 10 mg ml^−1^ caylase C4 (Cayla) (pH 5.8). The mycelia collected from a 125-ml overnight cultivation was incubated in the caylase C4 solution for ~ 3 h at 30 °C and 80 rpm. The resulting protoplast solution was filtered through two layers of Miracloth paper. The flowthrough was collected and protoplasts separated by centrifugation at 4 °C and 1500*g*. The separated protoplasts were washed with KMC and STC buffers using centrifugation for the separation. After the washes, protoplasts were resuspended in STC buffer. After the transformation reaction, standard 150 × 15 mm Petri dishes were used for plating.

### Screening of targeting efficiencies

In order to investigate the correct genome integrations, colony PCR using Phire direct PCR kit (Thermo) was used following the manufacturer’s protocol. Shortly, colonies were first collected from the transformation plates using pipette tips. Next, the collected mycelium was incubated for about 2 h in 20 µl of “dilution buffer” provided in the kit. PCR amplification of the target gene ORF and the junctions between the DSB ends and donor DNA cassette (deletion cassette or *UDH* expression cassette) were determined following the manufacturer’s instructions and using the primers listed in Additional file 1: Table S1. For the selected strains with two and three targets, the junctions between DSB ends and donor DNA were sequenced.

### Genomic copy number analysis

The mycelium for genomic DNA (gDNA) extraction was harvested by vacuum filtration. The gDNA was extracted by phenol chloroform method and were diluted 1:100 and 1:1000. The quantitative PCR (qPCR) analysis was carried out using a LightCycler II with the LightCycler SYBR green I Master mix (both Roche). DNA oligos oPEEL-373-380 were used in the analysis (Additional file 1: Table S1). The signal from target gene was normalized to actin using the accompanying software (Advance Relative Quantification tool).

### Chemical analyses

Samples containing mycelia were removed from liquid cultivations at intervals and were diluted as 1–5 (after 24 and 48 h) or as 1–10 (after 120 h) with 5 mM H_2_SO_4_. Diluted samples were heated at 100 °C for 1 h and filtered through 96-well Multiscreen HTS DV Filter Plate (0.65 µm Hydrophilic Low Protein Binding, Durapore^®^ Membrane). The concentration of galactarate was determined by HPLC using a Fast Acid Analysis Column (100 mm × 7.8 mm, BioRad Laboratories, Hercules, CA) linked to an Aminex HPX-87H organic acid analysis column (300 mm × 7.8 mm, BioRad Laboratories) with 5.0 mM H_2_SO_4_ as eluent and a flow rate of 0.5 ml min^−1^. The column was maintained at 55 °C. Peaks were detected using a Waters 2489 UV/Visible dual wavelength UV (210 nm) detector.

## Additional file

**Additional file 1.** Supplementary information.

## Abbreviations

CRISPR: clustered regularly interspaced short palindromic repeats
gRNA: guide RNA
sgRNA: single guide RNA
crRNA: CRISPR-RNA
tracrRNA: trans-activating crRNA
DSB: double-strand break
PAM: protospacer adjacent motif
NHEJ: non-homologous end joining
HDR: homology-directed repair
C-NHEJ: classical NHEJ
MMEJ: microhomology-mediated end joining
ssDNA: single-stranded DNA
RNP: ribonucleoprotein
HTP: high-throughput
MTP: microtiter plate
MoClo: modular cloning
CFU: colony forming unit
ORF: open reading frame
