# Supplementary material for: Development of microtiter plate scale CRISPR/Cas9 transformation method for Aspergillus niger based on in vitro assembled ribonucleoprotein complexes
Source: Fungal Biol Biotechnol. 2019 Mar 15;6:3. doi: 10.1186/s40694-019-0066-9 (PMC6419801; doi:10.1186/s40694-019-0066-9)
Supplement: Supplementary file 1 — Additional file 1. Supplementary information. [file 40694_2019_66_MOESM1_ESM.docx]

Additional File

**Table S1:** DNA oligos, crRNA protospacer sequences and *A. niger* strains used in this work.

|  | **DNA ID** | **Sequence** | **Description** |
| --- | --- | --- | --- |
| **Oligos for MoClo parts** | oPEEL-266 | GCATCGTCTCATCGGTCTCACAATCGAGTGCCTTTCTGGACTAT | 5' gaaX flanking seq, 1500 bp FWD |
|  | oPEEL-257 | GCATCGTCTCATCGGTCTCACAATATCGACAGCGAGTGAGATAT | 5' gaaX flanking seq, 1000 bp FWD |
|  | oPEEL-258 | GCATCGTCTCATCGGTCTCACAATTCAAGATCCTCTCTTCCTTG | 5' gaaX flanking seq, 500 bp FWD |
|  | oPEEL-259 | GCATCGTCTCATCGGTCTCACAATGATCTCTTCCTCCGCGATCG | 5' gaaX flanking seq, 100 bp FWD |
|  | oPEEL-260 | ATGCCGTCTCAGGTCTCAAGGGCAGTATACTGCTCGTTCTCA | 5' gaaX flanking seq 100-1500, REV |
|  | oPEEL-261 | GCATCGTCTCATCGGTCTCAGAGTTGAACTGTATCCACTCCTGG | 3' gaaX flanking seq, 100-1500 bp FWD |
|  | oPEEL-262 | ATGCCGTCTCAGGTCTCATCGGCCAGATTCTTGCAGGCGTTG | 3' gaaX flanking seq, 1500 bp REV |
|  | oPEEL-263 | ATGCCGTCTCAGGTCTCATCGGTTGCAGGCCTCACAGCTG | 3' gaaX flanking seq, 1000 bp REV |
|  | oPEEL-264 | ATGCCGTCTCAGGTCTCATCGGTGCCAGTCAGTAGTCGTCGT | 3' gaaX flanking seq, 500 bp REV |
|  | oPEEL-265 | ATGCCGTCTCAGGTCTCATCGGCTGTTGGATCATCACACTGC | 3' gaaX flanking seq, 100 bp REV |
|  | oPEEL-290 | GCATCGTCTCATCGGTCTCATACACAGGCTTGTCAGATATGTTC | A. oryzae PyrG to MoClo FWD |
|  | oPEEL-291 | ATGCCGTCTCAGGTCTCAACTCAAATGGTGGCTAGGCTCTGA | A. oryzae PyrG to MoClo REV |
|  | oPEEL-296 | GCATCGTCTCATCGGTCTCATATGTTGAGCTCCACCATGGCCT | UDH to moclo type 3 FWD |
|  | oPEEL-297 | ATGCCGTCTCAGGTCTCAGGATGGTTACTTATCGCCGAAGGG | UDH to moclo type 3 REV |
|  | oPEEL-311 | GCATCGTCTCATCGGTCTCACAATACAGCATCCAAGACTCACAT | 5' 39104 flanking seq, 1500 bp FWD |
|  | oPEEL-312 | ATGCCGTCTCAGGTCTCAAGGGAAACGCCCTACTATATCGGA | 5' 39104 flanking seq, 1500 bp REV |
|  | oPEEL-313 | GCATCGTCTCATCGGTCTCAGAGTGTAGGATTGAACAAGAGTGC | 3' 39104 flanking seq, 1300 bp FWD |
|  | oPEEL-314 | ATGCCGTCTCAGGTCTCATCGGTTGGAGTCGAAACCGTCCTC | 3' 39104 flanking seq, 1300 bp REV |
|  | oPEEL-322 | GCATCGTCTCATCGGTCTCAAACGTCAAGTGGGAGTTTGTGGAC | gaaB promoter to MoClo Type 2 FWD |
|  | oPEEL-324 | ATGCCGTCTCAGGTCTCACATAGGTGTCGGTTGTTTCTGTTC | gaaB promoter to MoClo Type 2 REV |
|  | oPEEL-325 | GCATCGTCTCATCGGTCTCACAATGCTATCGAGTTTATCACGGC | 5' gaaA flanking seq, 1500 bp FWD |
|  | oPEEL-326 | ATGCCGTCTCAGGTCTCAAGGGAGCCAGCGAACAATCCATTC | 5' gaaA flanking seq, 1500 bp REV |
|  | oPEEL-327 | GCATCGTCTCATCGGTCTCAGAGTTAATTGACAGGGGCGAACAA | 3' gaaA flanking seq, 1500 bp FWD |
|  | oPEEL-328 | ATGCCGTCTCAGGTCTCATCGGGGATCGAGGTAGGAGAACAC | 3' gaaA flanking seq, 1500 bp REV |
|  | oPEEL-352 | GCATCGTCTCATCGGTCTCACAATGGAAGGAGTGCAGAGTCGAG | 5' albA flanking seq, 1000 bp FWD |
|  | oPEEL-355 | ATGCCGTCTCAGGTCTCAAGGGGTTGAATTCAGCAGAGCAGC | 5' albA flanking seq, 1000 bp REV |
|  | oPEEL-356 | GCATCGTCTCATCGGTCTCAGAGTGTTATGCATTACGCCTTCCT | 3' albA flanking seq, 1000 bp FWD |
|  | oPEEL-358 | ATGCCGTCTCAGGTCTCATCGGTCTTTCCCTGTTCATACTAC | 3' albA flanking seq, 1000 bp REV |
| **Oligos for colony PCR** | oPEEL-279 | GGTGTCATTATTCGAAGTTC | gaaX deletion, colony PCR 5' region FWD |
|  | oPEEL-334 | GGCGAAAAAGGTATGGCAGA | gaaX deletion, colony PCR 3' region REV |
|  | oPEEL-292 | GAACATATCTGACAAGCCTG | Colony PCR, pyrG promoter REV |
|  | oPEEL-293 | TCAGAGCCTAGCCACCATTT | Colony PCR, pyrG promoter FWD |
|  | oPEEL-294 | GTTCTGGTCAACACGCATTG | gaaX ORF FWD |
|  | oPEEL-295 | CATGTTGACCGCTGAGAGGA | gaaX ORF REV |
|  | oPEEL-333 | CCACAATCTTCTTCTCGACG | gaaB checking protomoter |
|  | oPEEL-335 | CCTCACCACCGATCTCATTG | gaaA/gaaC deletion 5' region FWD |
|  | oPEEL-336 | GCTAGGGTGTTGTCCGTAAG | gaaA/gaaC deletion 3' region REV |
|  | oPEEL-337 | GACCTCCGACAAGAAAGTGG | gaaA ORF FWD |
|  | oPEEL-338 | TCTTGGGCTGACTCATGTAG | gaaA ORF REV |
|  | oPEEL-339 | GACCAGAGTTGACGACGATA | 39114 deletion 5' region FWD |
|  | oPEEL-340 | GGAATCCTCATCCTCATCGT | 39114 deletion 3' region REV |
|  | oPEEL-341 | TCGGACAGCTCTCAAAGAAC | 39114 ORF FWD |
|  | oPEEL-342 | AAGAGTTCTGTGCGAGTTGG | 39114 ORF REV |
| **Oligos for qPCR** | oPEEL-373 | CCGTCACCATGGGTAAAGCT | reference gene actin version 1 FWD |
|  | oPEEL-374 | TGGGGTATCTGAGGGTGAGG | reference gene actin version 1 REV |
|  | oPEEL-375 | CCCTCTGGCGCTTATGGAAA | reference gene actin version 2 FWD |
|  | oPEEL-376 | GCACAGCGGATATGAGGAGG | reference gene actin version 2 REV |
|  | oPEEL-377 | CATGATGTCCACCTGGCTGT | target gene UDH version 1 FWD |
|  | oPEEL-378 | AATCCTTGGGGGCGTAATCG | target gene UDH version 1 REV |
|  | oPEEL-379 | GGAAGTCCAGGTCTGCGATC | target gene UDH version 2 FWD |
|  | oPEEL-380 | CGGCCTCGTAGATGTGGAAC | target gene UDH version 2 REV |
|  | **crRNA ID** | **crRNA protospacer sequence** | **Target gene** |
| **Protospacer sequences** | gERA-001 | GTGGAAGTGGACGTCGCCAG | gaaX |
|  | gERA-002 | GGTGATTGGCGGGTGAACGG | gaaX |
|  | gERA-009 | GCGTAAACCTCTTCAATCAG | gaaA-gaaC |
|  | gERA-010 | GGCTTCTCCGGACTGCACAG | gaaA-gaaC |
|  | gERA-015 | CTGAGTACGAAGGGAAACGA | 39114 |
|  | gERA-016 | AAACTGACCGAGAGAAACCA | 39114 |
|  | gERA-017 | AGTGGGATCTCAAGAACTAC | albA |
|  |  | **Strain name / genotype** | **Description** |
| **A. niger strains** |  | *∆pyrG* | Pyrimidine-deficient *A. niger* ATCC105 |
|  |  | M1767 / *ΔgaaA-Δ39114-UDH* | Previously described galactarate producing strain, disrupted  galacturonate reductase, gaaA, and the galactarate catabolism gene, 39114, introduced bacterial uronate dehydrogenase, UDH under gpdA promoter. |
|  |  | *ΔgaaA-ΔgaaC_0-264bp_-UDH* | Strain generated in this study using single-taget CRISPR/Cas9,  disrupted galacturonate reductase, gaaA, and  2‐keto‐3‐deoxy‐L‐galactonate aldolase, gaaC, introduced bacterial uronate dehydrogenase, UDH, under gaaB promoter. |
|  |  | *ΔgaaA-ΔgaaC_0-264bp_-Δ39114-UDH* | Strain generated in this study using two-taget CRISPR, disrupted galacturonate reductase, gaaA, 2‐keto‐3‐deoxy‐L‐galactonate aldolase, gaaC,  and galactarate catabolism gene 39114, introduced bacterial uronate dehydrogenase, UDH, under gaaB promoter. |
|  |  | *ΔgaaA-ΔgaaC_0-264bp_-Δ39114-ΔgaaX-UDH* | Strain generated in this study using three-taget CRISPR, disrupted galacturonate reductase, gaaA, 2‐keto‐3‐deoxy‐L‐galactonate aldolase, gaaC, the galactarate catabolism gene 39114, and the regulatory protein gaaX,  introduced bacterial uronate dehydrogenase, UDH, under gaaB promoter. |


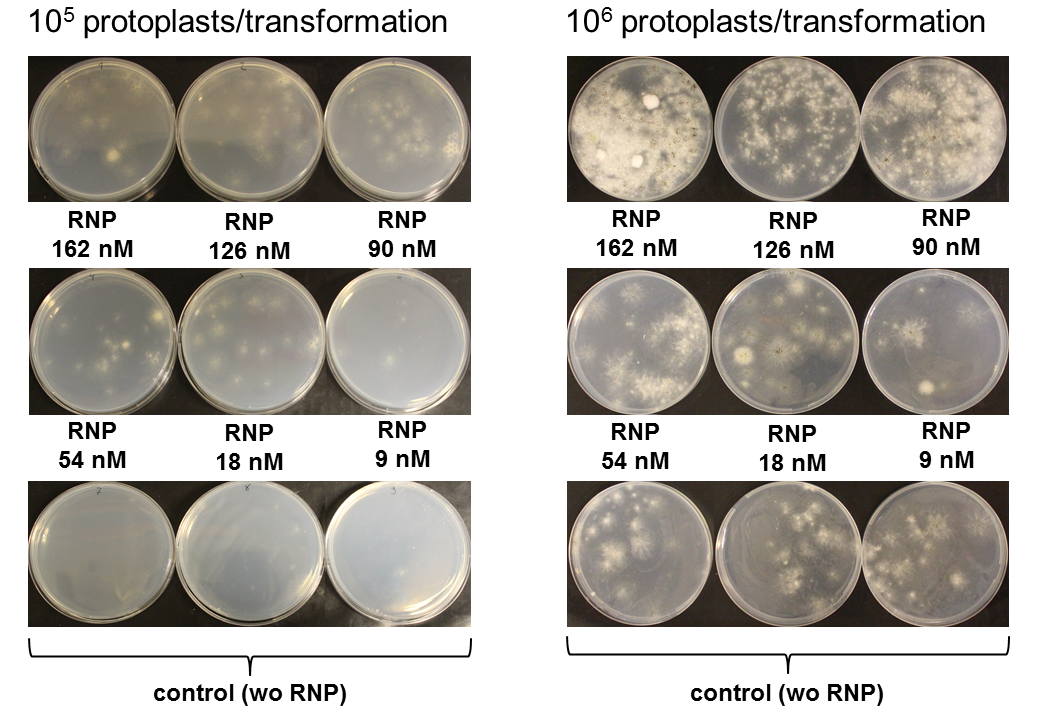


**Figure S1:** Transformation plates of *gaaX* deletion in *A. niger* using 1 µg of donor DNA, different concentrations of RNP complex and 10^5^ or 10^6^ protoplasts per transformation.


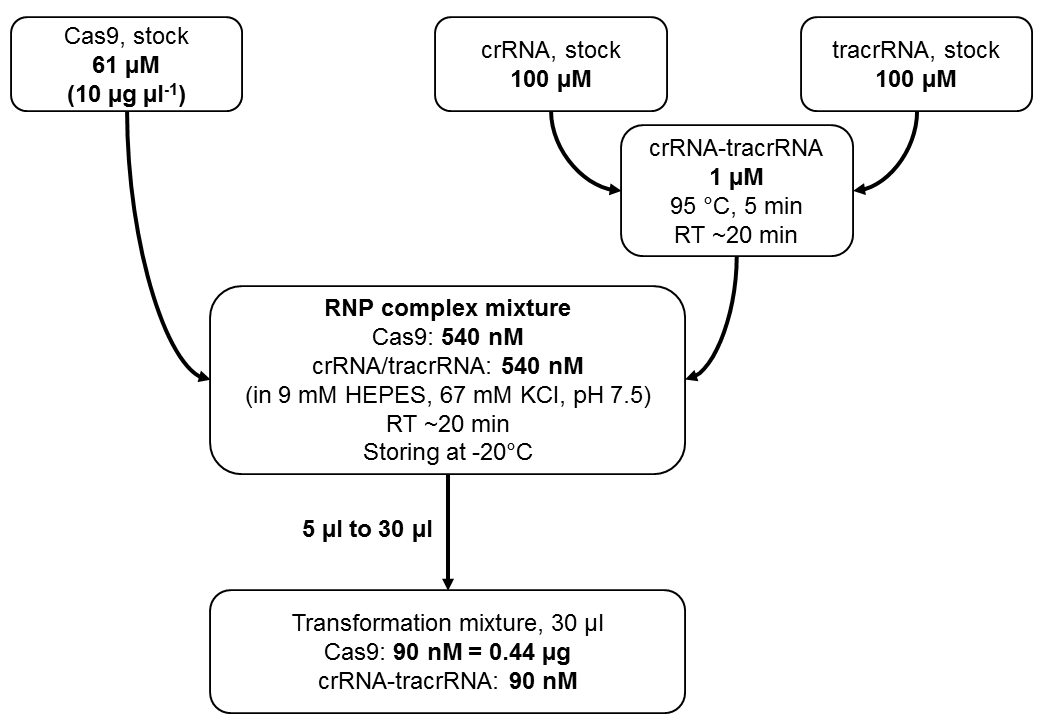


**Figure S2:** Stock solutions and dilutions of Cas9, crRNA and tracrRNA for RNP complex preparation and in transformation.


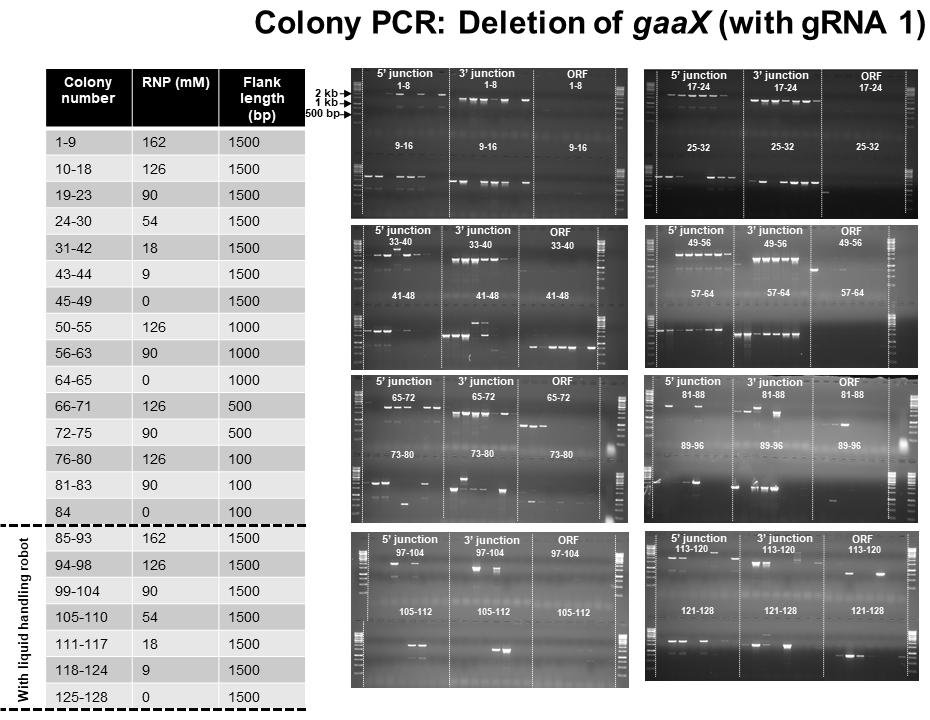


**Figure S3:** Agarose gel pictures of colony PCR reactions detecting replacement of *gaaX* with the deletion cassette. Junctions between the donor DNAs and double-strand brakes at the 5’ (5’ junctions, oPEEL-279/-292) and 3’ (3’ junctions, oPEEL-293/-334) ends were analysed. In addition, the absence of *gaaX* open reading frame was confirmed (ORF, oPEEL-294/-295).

**
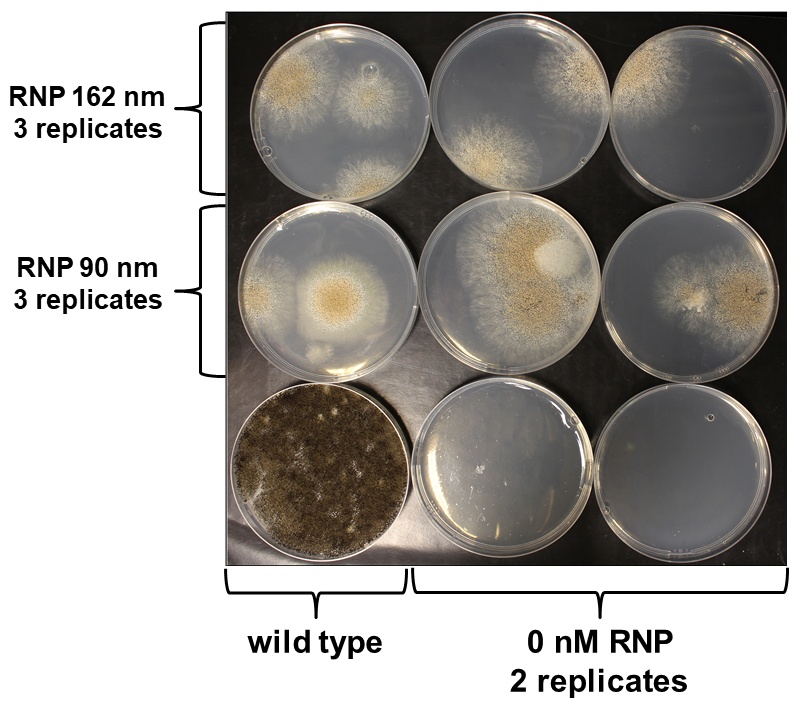
**

**Figure S4:** Transformation plates of *albA* deletion in *A. niger* using 162, 90 or 0 mM RNP complex, 1 µg of donor DNA with 1000 bp flanking sequences (also with 0 nM RNP) and 10^5^ protoplasts per transformation. Correct deletion of *albA* results in disruption of conidia pigment formation.


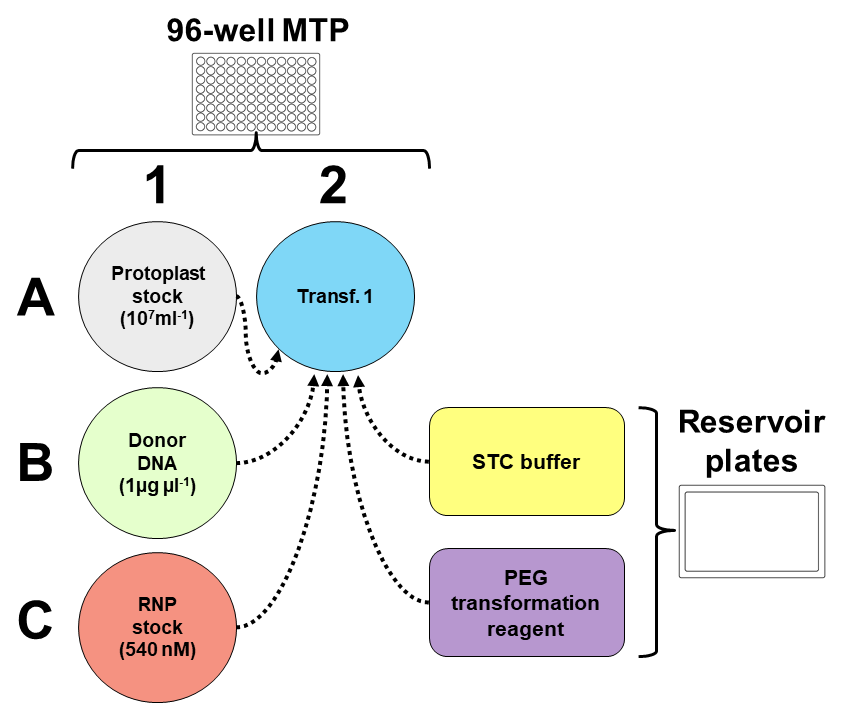


**Figure S5:** The pipetting workflow with the liquid handling robot. Circles represent microtiter plate wells and rounded rectangles reservoir plates.

**
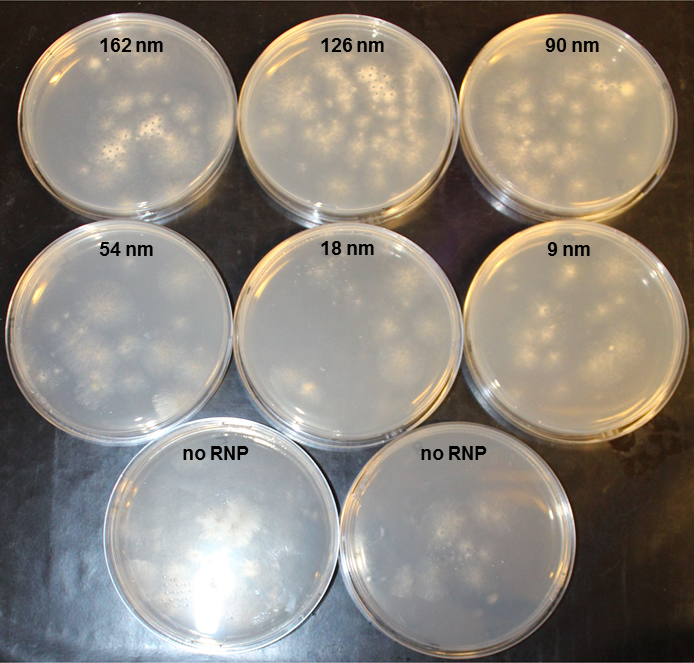
**

**Figure S6:** Transformation plates resulting from liquid handling robot facilitated transformation reactions for *gaaX* deletion in *A. niger* using 1 µg of donor DNA with 1500 bp flanking sequences, different concentrations of RNP complex and 10^5^ protoplasts per transformation.

**
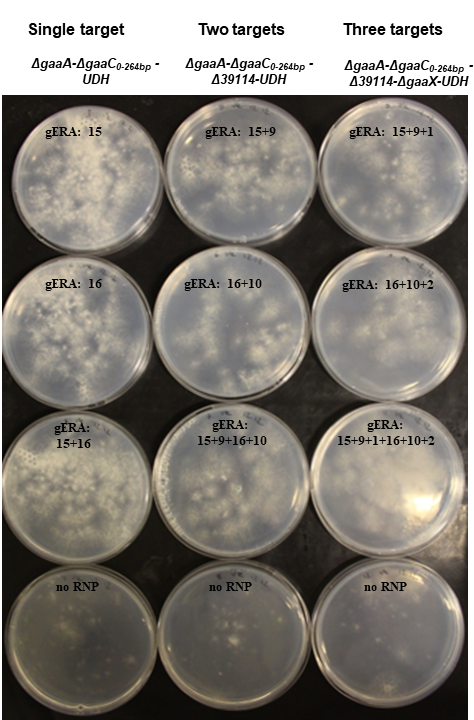
**

**Figure S7:** CRISPR/Cas9 transformation plates of engineered *A. niger* strains for galactarate production. Single (in *ΔgaaA-ΔgaaC_0-264bp_-UDH* strain), two (in *ΔgaaA-ΔgaaC_0-264bp_-Δ39114-UDH* strain) or three (in *ΔgaaA-ΔgaaC_0-264bp_-Δ39114-ΔgaaX-UDH* strain) genomic targets were replaced with the UDH expression cassette. The RNP complex concentration of 90 nM and 1 µg of donor DNA with 1500 bp flanking sequences were used for each of the genomic targets. The protoplast concentration 10^5^ per transformation was used in all of the transformations. gERA numbers (sequences presented in Additional File: Table S1) represent different crRNA combinations used in the RNPs. When two different crRNAs for a single genomic target was used (gERA: 15+9+16+10 and gERA: 15+9+1+16+10+2), the concentration of each of the RNPs was 45 nM.


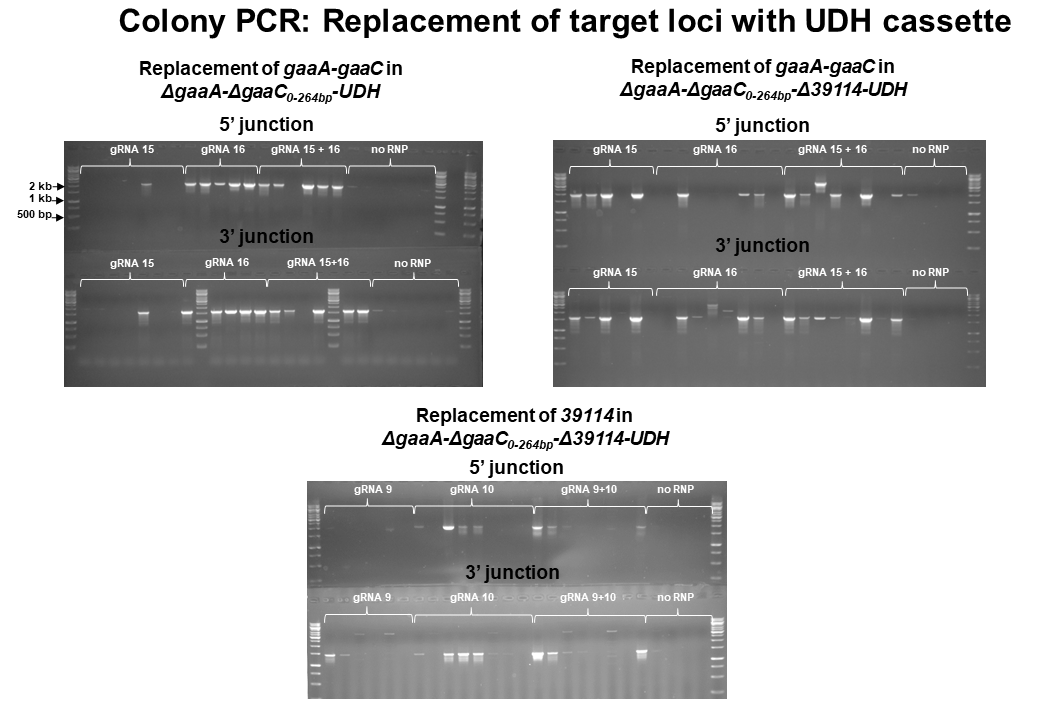


**Figure S8:** Agarose gel pictures of colony PCR reactions detecting replacement of *gaaA-gaaC* and *39114* with the *UDH* expression cassette in the multiplexed CRISPR/Cas9 transformations for two target loci. Junctions between the donor DNAs and double-strand brakes at the 5’ (5’ junctions, at *gaaA-gaaC* oPEEL-335/-333, at *39114* oPEEL-339/-333) and 3’ (3’ junctions, at *gaaA-gaaC* oPEEL-293/-336, at *39114* oPEEL-293/-340) ends were analysed.


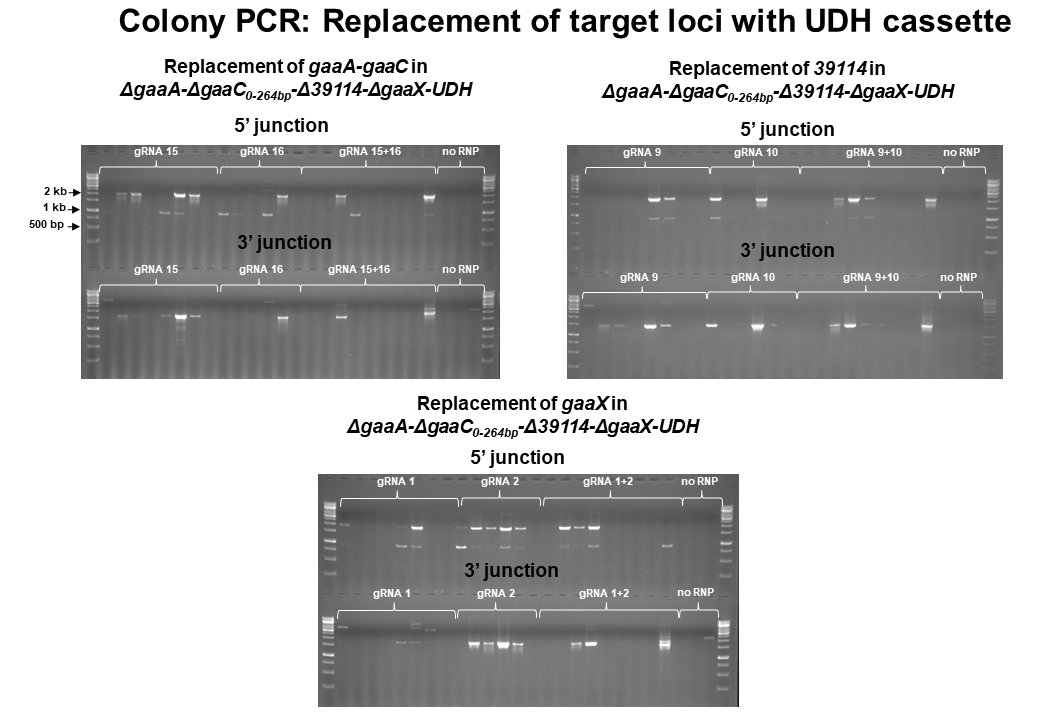


**Figure S9:** Agarose gel pictures of colony PCR reactions detecting replacement of *gaaA-gaaC 39114* and *gaaX* with the *UDH* expression cassette in the multiplexed CRISPR/Cas9 transformations for three target loci. Junctions between the donor DNAs and double-strand brakes at the 5’ (5’ junctions, at *gaaA-gaaC* oPEEL-335/-333, at *39114* oPEEL-339/-333, at *gaaX* oPEEL-279/-333) and 3’ (3’ junctions, at *gaaA-gaaC* oPEEL-293/-336, at *39114* oPEEL-293/-340, at *gaaX* oPEEL-293/-334) ends were analysed.


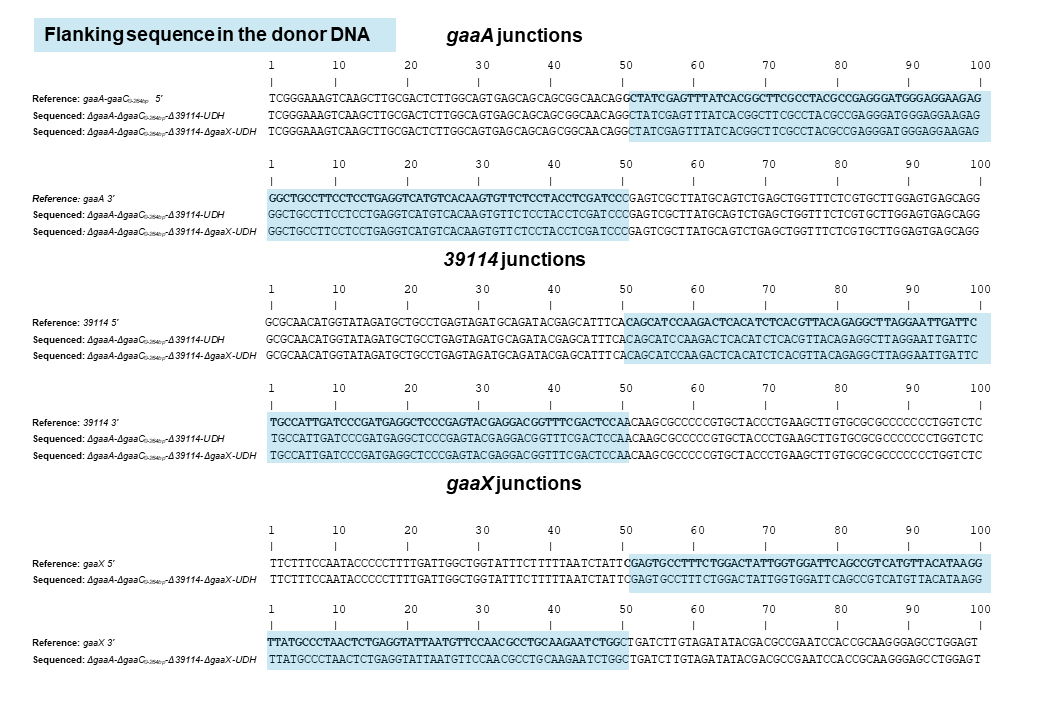


**Figure S10:** Genomic DNA sequence analysis of the genomic junctions between the flanking sequences in the donor DNA cassettes and target loci in the engineered *A. niger* strains after multiplexed CRISPR/Cas9 genome editing.

**Figure S11:** qPCR genomic copy number analysis of the UDH cassettes in the strains *ΔgaaA-ΔgaaC_0-264bp_ -UDH* (yellow, number 1), *ΔgaaA-ΔgaaC_0-264bp_-Δ39114 -UDH* (orange, number 2) and *ΔgaaA-ΔgaaC_0-264bp_-Δ39114-ΔgaaX-UDH* (blue, number 3). Oligos oPEEL-373-380 were used in the analysis. Values represent the means of 24 technical replicates ± standard deviation.
